# Thinking Outside the Box

**DOI:** 10.7759/cureus.20970

**Published:** 2022-01-05

**Authors:** Sara Trevas, Renato Guerreiro, Catarina Faria, Ana Santos

**Affiliations:** 1 Internal Medicine Department, Hospital São Francisco Xavier, Lisbon, PRT

**Keywords:** hypertension, fever, seizures, diagnostic errors, bias, clinical decision making

## Abstract

Clinical decision-making process is very complex and influenced by multiple aspects. As diagnosis likelihood assessment is often based on intuitive thinking, data misinterpretation, and diagnostic errors may commonly occur. We present a peculiar clinical case of a 27-year-old obese woman admitted to the emergency department after an inaugural episode of seizures. She had an oncologic disease. She was febrile and hypertensive at first evaluation. The report evolves around the diagnostic assessment, hampered by incongruent anamneses, incorrect data interpretation, and a pinch of clinical obstination, which nearly culminated in two deaths. Then, we discuss the series of biases that have confused the physicians. The only way to escape the intuitive thinking trap is to be humbly aware of our own thinking method’s limitations and to learn about the biases that often lead us into errors. Sometimes, thinking outside the box is the key.

## Introduction

Clinical decision-making is a complex process. It involves gathering data, interpreting the collected information, determining probabilities, and repetition of these steps until the diagnostic decision is made [1]. Many aspects influence this exercise. For example, in the setting of the emergency department, collecting clinical data could be challenging, especially when the patient is unable to give a proper or reliable history and/or is not cooperating with physical examination. Moreover, the diagnosis likelihood assessment is often based on intuitive thinking, which is fast and convenient but could result in data misinterpretation [2]. In this article, we present a peculiar clinical case in which distinct factors contributed to delays in the correct diagnosis and treatment establishment.

## Case presentation

A 27-year-old woman was admitted to the emergency department after an inaugural episode of seizures. At her first clinical evaluation, she said she had been having tension headaches and phonophobia for the last three days, but no other symptoms. She stated she was an oncologic patient: as she said, “both colon and uterine cancer” and “undergoing chemo and radiotherapy.” To her knowledge, there weren't any cerebral metastases. Since her follow-up was being conducted by a different health center, there weren't any available electronic medical reports to consult. Additionally, she denied other known medical issues.

At that moment, the patient was febrile (38.0ºC) and hypertensive (200/170 mm Hg), but the revolving clinical exam was said to be “normal”-including a Glasgow Coma Scale of 15 points, isochoric and isoreactive pupils, no neck stiffness, absence of Brudzinski’s and Kerning’s signs, and no focal neurological deficits. She was an obese woman (body mass index of 31.2 kg/m^2^). While being examined, the patient had two generalized tonic-clonic seizure episodes. After the second one, she didn't fully recover and she was intubated for a secure airway. It was also noticed a vesical globe, so a urinary catheter was placed.

Several exams were performed. The arterial blood gas analysis showed metabolic acidosis with hyperlactacidemia (pH 7.25, PaCO_2_ 22 mm Hg, PaO_2_ 110 mm Hg, HCO_3- _9.3 mmol/l, SaO_2_ 98%, lactate 10.3 mmol/l, glucose 101 mg/dl). The blood test revealed leukocytosis with neutrophilia without renal, hepatic, thyroid function impairment, or electrolyte imbalance (Table 1). The toxicological screening tests were negative, as was the preliminary infectious diseases assessment (Table 2). The cranial computed tomography scan was normal. Lastly, a lumbar puncture was performed, showing a turbid cerebrospinal fluid (CSF) with high protein, low glucose content, and polymorphonucleocyte predominance despite low cell count (Table 3).

**Table 1:** Blood test analysis.

Parameter	Result	Reference range
Hemoglobin (g/dl)	13.0	12.0-15.0
Leukocyte count (×10^9^/l)	22.0	4.0-10.0
Platelet count (×10^9^/l)	415	150-400
D-dimer (ng/ml)	2,087	0-500
Glucose (mg/dl)	105	74-106
Albumin (g/dl)	4.2	3.5-5.2
Bilirubin (mg/dl)	0.9	<1.4
Aspartate aminotransferase (U/l)	12	<40
Alanine aminotransferase (U/l)	11	<41
Alkaline phosphatase (U/l)	35	40-130
Gamma-glutamyl transferase (U/l)	28	10-71
Urea (mg/dl)	35	17-49
Creatinine (mg/dl)	0.9	0.7-1.2
Sodium (mmol/l)	140	135-145
Potassium (mmol/l)	4.3	3.5-5.10
Chloride (mmol/l)	99	98-107
Phosphorus (mg/dl)	2.90	2.5-4.5
Magnesium (mg/dl)	1.7	1.6-2.4
Calcium (mg/dl)	9.1	8.8-10.2
Creatine kinase (U/l)	110	25-200
High sensitivity troponin T (ng/l)	13	<14
C-reactive protein (mg/dl)	1.30	<0.5
Thyroid stimulating hormone (mU/l)	3.99	0.45-4.5

 

**Table 2:** First toxicological and infectious diseases screening.

Exam	Result
Toxicological screening	Negative for valproic acid, tricyclics, barbiturates, acetaminophen, benzodiazepines, ethanol, opiates, cocaine, cannabinoids, and amphetamines
Serology tests	Negative for human immunodeficiency virus, hepatitis B virus, hepatitis C virus, Epstein-Barr virus, cytomegalovirus, and *Treponema pallidum*
Urinary antigens	Negative for* Streptococcus pneumoniae* and *Legionella pneumophila*
Nasopharyngeal swab (polymerase chain reaction tests)	Negative for SARS-CoV-2, influenza virus A and B, and respiratory syncytial virus

**Table 3:** Cerebrospinal fluid analysis.

Parameter	Result	Reference range
Opening pressure (mmH_2_0)	Not measured	50-200
Color	Xanthochromic but with clear supernatant after centrifugation	Crystal clear
Leukocyte count (cells/ul)	4.0	0-5.0
Leukocyte type predominance	Polymorphonucleocytes	Lymphocytes
Glucose (mg/dl)	49	50-80
Proteins (mg/dl)	63	15-40
Gram stain	Negative	Negative
Antigen tests	Negative for *Haemophilus influenzae type b*, *Neisseria meningitidis*, *S. pneumoniae*, *Cryptococcus neoformans,* and *C. gattii*	Negative

At this point, the assumed diagnosis was a refractory status epilepticus in the onset of an acute central nervous system infection-meningitis (either bacterial or viral, yet to be discovered). An empiric combination of vancomycin, ceftriaxone, acyclovir, and dexamethasone was initiated, along with levetiracetam and sodium valproate.

The patient was transferred for the intensive care unit (ICU).

In the first hours of admission, she became hypoxemic. Therefore, a computed tomography pulmonary angiogram was performed, excluding pulmonary emboli. However, it revealed bilateral basal lung consolidations of likely infectious cause-a community-acquired pneumonia was also assumed. Other complementary exams were performed but inconclusive (Table 4).

**Table 4:** Complementary diagnostic exams.

Exam	Result
Cultural exams	Negative blood, urine, cerebrospinal fluid, and bronchoalveolar lavage fluid cultures
Galactomannan test	Negative both in serum and bronchoalveolar lavage fluid
Cerebrospinal fluid polymerase chain reaction tests	Negative for cytomegalovirus, Epstein-Barr virus, enterovirus, adenovirus, BK polyomavirus (BKPyV) and JC polyomavirus (JCPyV), influenza virus A and B, human herpesvirus 6, herpes virus simplex 1 and 2, varicella zoster virus, *Haemophilus influenzae*, *Neisseria meningitidis*, *S. pneumoniae*, *Mycoplasma pneumoniae*, *Borrelia* spp., *Cryptococcus neoformans*, *Toxoplasma gondii*, *Listeria monocytogenes*, *Streptococcus agalactiae,* and *Mycobacterium tuberculosis*
Bronchoalveolar lavage fluid polymerase chain reaction test	Negative for SARS-CoV-2
Interferon gamma release assay	Negative
Echocardiography	Normal size of ventricles and atria. Normal left ventricular ejection fraction. No wall motion abnormalities. No pericardial effusion.
Second cranial computed tomography scan (24 h apart from first one)	Normal
Computed tomography cerebral venography scan	Normal

After 24 hours of ICU admission, despite the down-titration of sedatives, the patient hasn’t recovered consciousness. The electroencephalogram didn't report any paroxysmal activity, and the remaining neurological evaluation was normal. In addition, she remained hypertensive, even under multiple antihypertensive drugs (labetalol, amlodipine, and captopril).

Due to a lack of background information regarding the patient's medical history, her boyfriend and family were questioned about it. They confirmed the patient’s cancer statement, but they didn’t know any details of her medical status since she “preferred to go alone” to all medical/treatment appointments. The proclaimed health center she frequented was contacted: there weren’t any records in this patient’s name and there wasn’t even an oncology department in its facilities.

Finally, as part of the complementary study of the comatose state and hypertension, an immunology pregnancy test was preformed, and it was positive. The serum beta-human chorionic gonadotropin dosing (8739 mIU/ml) and obstetric echography confirmed the pregnancy. The suprapubic ultrasound disclosed a 25-26 week gestation and oligohydramnios. The urinalysis showed proteinuria, which, along with hypertension and seizures, supported the plausible diagnosis of eclampsia. As such, an emergency cesarean was performed, and an extremely low-weight (800 g) female baby was delivered and shifted to the incubation bed in the neonatal ICU.

After giving birth, the patient's condition rapidly improved. She was extubated hours later and fully recovered consciousness. Furthermore, half of the antihypertensive drugs were stopped, and no more seizures were observed during the stay.

Lastly, the patient was evaluated by the psychiatric team. When confronted with her current medical situation, she calmly confessed she had been faking the cancer diagnosis for five years and lying to her family about it; she also claimed that she “didn’t notice” the pregnancy and was “happily surprised” about it, showing an attitude we interpreted as la belle indifférence. Subsequently, she was diagnosed with factitious disorder and cluster B personality traits.

After two days of clinical stability in the ICU, the patient was transferred to the medicine ward and discharged a week later, presenting no sequels whatsoever.

The baby had an omphalocele and underwent several corrective surgical procedures, but also survived.

## Discussion

Eclampsia is a severe complication of preeclampsia, manifested by the new onset of generalized tonic-clonic seizures and/or coma. Preeclampsia is defined as the new onset of hypertension after 20 weeks of gestation with proteinuria and/or end-organ dysfunction. The incidence of eclampsia is low (1.5-10 per 10,000 deliveries). The diagnosis is clinical, and the management includes antihypertensives and prevention of recurrent seizures with magnesium sulfate. Nevertheless, the curative approach is prompt delivery. This condition is associated with high morbidity and mortality of both the mother and fetus, so a quick and proper diagnosis is critical [3,4].

Obviously, the first step in recognising an eclampsia is to recognise the pregnancy, which in this case report was not linear. There were multiple diagnostic errors along the way.

It’s estimated that the diagnostic failure rate is 10% to 15% in clinical practice. Diagnostic errors usually do not happen because of a lack of scientific knowledge but because of impaired physicians thinking instead. As part of the intuitive thinking process, humans use mental shortcuts to make easier and faster decisions. However, this method is susceptible to disrupted perceptions, also known as cognitive biases [2,5].

In this case report, there were plenty of biases involved. Initially, despite poor detailed data, the physicians believed the patient had an oncological disease (information bias). Therefore, this has driven to the enhancement of cancer-related hypotheses, such as infections, thrombotic/thromboembolic events, and cerebral metastases (attentional and attribution biases). Although there was incongruent data supporting this theory-as so: the presence of fever and leucocytosis but normal CSF analysis, normal neuroimaging exams, and negative infectious tests-it was assumed the patient had meningitis (anchoring bias). There were discarded other plausible etiologies-toxins, electrolyte disturbance, thyroid malfunction-creating a false sense of security that it certainly wasn’t something else (confirmation and diagnosis momentum biases). Since the patient was obese and the pregnancy complicated with oligohydramnios, there wasn’t a noticeable abdominal prominence and even the abdominal uterus palpation was mistaken for a vesical globe (ascertainment bias and framing effect). Also, due to the belief that the patient was undertaking chemo and radiotherapy, a pregnancy seemed implausible (prototypical error and zebra retreat biases). Only later, when the patient continued comatose and hypertensive- and when the cancer diagnosis dropped (due to the crescent history incongruences)-an eclampsia on the site of a cryptic pregnancy strike as an attainable diagnosis. In other words, once the model of thinking shifted to an analytic and wider form, new hypotheses were raised and the diagnosis was possible [6].

Nevertheless, it’s important to notice that this is an unusual mixture of events. Eclampsia is an uncommon condition, as it is a cryptic pregnancy or factitious disorder.

Cryptic pregnancies could be concealed pregnancies (in which the woman knows she is pregnant but hides the pregnancy from everyone) or denied pregnancies (when the woman is unaware, she is pregnant or unable to accept it). The prevalence is unknown; it has been estimated that it occurs in one per 475 pregnancies. Many factors could contribute to it: absence of traditional physical symptoms of pregnancy, inexperience, external stress, fear of disapproval, cognitive impairment, or even psychotic illness. In this case report, the patient was diagnosed with factitious disorder, underlying possible psychological internal conflicts that led to either denial or concealing the pregnancy from doctors and family and not seeking medical follow-up [7,8].

Factitious disorder (also known as Munchausen syndrome) is a severe mental disorder in which someone intentionally falsifies physical and/or mental signs and symptoms in themselves or others, for no apparent external gain or reward. The prevalence of this illness is unknown due to difficulty in obtaining data (because of its dishonest nature). In this case report, this diagnosis was suspected because of conflicting medical history details. Then it was confirmed after a thorough psychiatric evaluation [9]. 

## Conclusions

It is very easy to misjudge a situation when given misleading information. It is, also, very easy to ignore alternative and less common conditions-of course, when you hear hoofbeats, think horses not zebras. But sometimes, switching the decision-making process from an intuitive form to a more analytical/mindful manner (i.e., thinking outside the box) is the key to solve the case. The only way to escape the intuitive thinking trap is to be humbly aware of our own thinking method’s limitations and to learn about the biases that often lead us into diagnostic errors. In this clinical case, there were present three uncommon disorders at once, and the physicians had to overcome a series of clinical biases to finally discover them. Luckily, at the end, two lives were saved.
